# Genome Evolution and the Emergence of Fruiting Body Development in *Myxococcus xanthus*

**DOI:** 10.1371/journal.pone.0001329

**Published:** 2007-12-26

**Authors:** Barry Goldman, Swapna Bhat, Lawrence J. Shimkets

**Affiliations:** 1 Applied Bioinformatics, Monsanto Company, St. Louis, Missouri, United States of America; 2 Department of Microbiology, University of Georgia, Athens, Georgia, United States of America; Baylor College of Medicine, United States of America

## Abstract

**Background:**

Lateral gene transfer (LGT) is thought to promote speciation in bacteria, though well-defined examples have not been put forward.

**Methodology/Principle Findings:**

We examined the evolutionary history of the genes essential for a trait that defines a phylogenetic order, namely fruiting body development of the Myxococcales. Seventy-eight genes that are essential for *Myxococcus xanthus* development were examined for LGT. About 73% of the genes exhibit a phylogeny similar to that of the 16S rDNA gene and a codon bias consistent with other *M. xanthus* genes suggesting vertical transmission. About 22% have an altered codon bias and/or phylogeny suggestive of LGT. The remaining 5% are unique. Genes encoding signal production and sensory transduction were more likely to be transmitted vertically with clear examples of duplication and divergence into multigene families. Genes encoding metabolic enzymes were frequently acquired by LGT. Myxobacteria exhibit aerobic respiration unlike most of the δ Proteobacteria. *M. xanthus* contains a unique electron transport pathway shaped by LGT of genes for succinate dehydrogenase and three cytochrome oxidase complexes.

**Conclusions/Significance:**

Fruiting body development depends on genes acquired by LGT, particularly those involved in polysaccharide production. We suggest that aerobic growth fostered innovation necessary for development by allowing myxobacteria access to a different gene pool from anaerobic members of the δ Proteobacteria. Habitat destruction and loss of species diversity could restrict the evolution of new bacterial groups by limiting the size of the prospective gene pool.

## Introduction

Speciation in higher organisms usually occurs in genetic isolation. Successive rounds of gene duplication and divergence, followed by individual gene loss, is thought to have contributed to morphological diversification [1]. Speciation has not been as extensively studied in bacteria. Small gene duplication events have been noted in many sequenced bacterial genomes [2] but lateral gene transfer (LGT) also contributes to genome diversification. Bacteria exchange genes by conjugation, transformation, and transduction which are widespread in nature and can occur between distantly related organisms [3]. Genome sequencing has revealed substantial rates of foreign gene acquisition [4]. Analysis of all gene phylogenies in the sequenced members of the γ Proteobacteria indicated that LGT rather than gene duplication provided most of the diversity in genomic repertoires [5]. LGT also has the potential to drive metabolic innovation. The dissemination of antibiotic resistance genes on a global scale is the paradigm, but examples of disseminated gene clusters for metabolic pathways and pathogenicity determinants also exist [6], [7]. These results provide compelling evidence that LGT can influence genotype evolution at the level of sub-speciation and by extension LGT may aid bacterial speciation.

While gene duplication in *M. xanthus* led to the production of several large multi-gene families [8], the role of LGT in *M. xanthus* genome evolution has not been evaluated. LGT is generally supported if a phylogenetic tree for a gene is in disagreement with that of the 16S rDNA gene, although incongruent trees can also arise by gene loss from related lineages. The other method to detect LGT relies on differences in the nucleotide sequence composition of a gene relative to average of that particular host. Given enough time acquired genes converge in codon usage with the bulk of the genome by amelioration [9], which make ancient transfers difficult to detect with compositional algorithms. Furthermore, LGT may be most successful when foreign genes and recipient genomes have similar codon usage [10]. Nevertheless, genes with codon usage that differs from that of the host are excellent candidates for LGT [11], [12].

Prokaryotic evolution is the product of environmental pressures combined with changes in the gene repertoire over time. The myxobacteria provide an opportunity to examine genome evolution within the context of speciation because fruiting body development is a unique trait that defines a phylogenetic order. In this work we examined the evolutionary history of *M. xanthus* genes required for fruiting body development using compositional and BLAST algorithms. Our results suggest that genes for metabolic enzymes, particularly those involved in polysaccharide biosynthesis, were more likely to be acquired by LGT and genes for sensory systems were more likely to be vertically inherited with numerous examples of duplication and divergence.

## Results

*M. xanthus* and other myxobacteria are members of the δ Proteobacteria, one of the most diverse groups of Bacteria in terms of habitat distribution and respiration strategies. Hundreds of δ Proteobacteria species are known, but for the sake of brevity, only those genera with a complete genome sequence will be mentioned here. With the exception of the myxobacteria and *Bdellovibrio* most of the genera, including *Anaeromyxobacter, Desulfotalea, Desulfovibrio, Geobacter, Lawsonia, Pelobacter,* and *Syntrophus* are anaerobic (use terminal electron acceptors other than O_2_). Many are also chemolithotrophs (use inorganic compounds as energy sources) and autotrophs (derive all their carbon from CO_2_). While the *modus operandi* for most genera in the δ Proteobacteria is metabolic diversity, the myxobacteria are neither chemolithotrophs nor autotrophs and instead devote their resources to social cooperativity directed toward predation and the construction of a unique multicellular structure, the asexual fruiting body.

A phylogenetic approach was initially used to identify possible cases of LGT in *M. xanthus*. Similarity searches using the BLASTP algorithm, with a 10^−10^ cutoff, were performed with each putative *M. xanthus* protein. The top four BLASTP hits were used because the top hit does not always represent the closest homolog and more distant hits can be widely diverged from the *M. xanthus* query sequence. One or more of the top four hits was a δ Proteobacteria for 55.0% of the genes suggesting a vertical descent as the inheritance paradigm for over half of the *M. xanthus* genes. Genomes also contain unique genes, in this case 22.2%, that have not been found in any other organism (so called ORFans). The remaining gene products (22.8%) have no homologs among δ Proteobacteria in the top 4 BLASTP hits, but often striking homology with gene products from other organisms suggestive of an alien origin.

The same phylogenetic analysis was applied to other members of the δ Proteobacteria. With the exception of *Bdellovibrio*, which contains 43.0% δ Proteobacteria genes, other genera in the δ Proteobacteria contained a higher proportion of δ Proteobacteria genes, including *Anaeromyxobacter* (60.7%), *Desulfovibrio* (84.1%), *Geobacter* (90.2%), *Lawsonia* (83.6%), and *Pelobacter* (74.7%). *Bdellovibrio* is thought to have acquired an unusually large number of genes via LGT [13]. These results suggest that *M. xanthus* has received more alien genes by LGT than most other δ Proteobacteria.

### Fruiting body development

The myxobacteria represent an evolutionary branch that culminated in a unique and striking form of multicellularity. The evolutionary history of genes known to be required for fruiting body development was examined in order to glean some insight into principles guiding the evolution of this group of organisms. Only genes in which mutations diminish the capacity to form fruiting bodies or cause at least a 10-fold decrease in sporulation are included here. Many genes that affect the timing of development, but not the integrity of the end product, have been reported but were not considered here.

The majority of the genes examined in this study have been published previously and are the subject of ongoing investigations into the mechanism of fruiting body development. This group of genes was supplemented with developmental mutants identified using the mariner-based transposable element *magellan-4* [14], which was used previously to identify motility genes in *M. xanthus* [15]–[17]. 40,000 *magellan-4* insertions were screened for loss of fruiting body development. Mutant genes were backcrossed into the wild type DK1622 to confirm that the transposon causes the mutant phenotype. These genes, together with previously identified developmental genes, are given in table 1. The phylogeny of each gene was examined using the BLASTP algorithm. Genes with a codon usage that differed from that of the majority of the host genes were identified with software developed by Karlin and Mrazek [11], [12].

**Table 1:** Genes required for *M. xanthus* fruiting body development.

MXAN Locus Tag	Protein Product	Function	Codon Bias^1^	Phylogeny^2^	Code^3^
0581	InfC	Translation initiation factor	0.038	Delta Proteobacteria	S
0733	RodK	Sensor histidine kinase		Delta Proteobacteria	S
1014	SdeK	Sensor histidine kinase		Delta Proteobacteria	S
1020		Hypothetical		Delta Proteobacteria	
1078	Nla19	Sigma 54-dependent response regulator		Delta Proteobacteria	S
1167	Nla28	Sigma 54 dependent response regulator		Delta Proteobacteria	S
1294	CsgA	C-signal		Delta Proteobacteria	S
1402	LadA	Transcription factor		Actinobacteria	S
				Planctomyces	
1450	Oar	TonB-dependent receptor		Delta Proteobacteria	S
2044	Pph1	Ser/Thr protein phosphatase		Delta Proteobacteria	S
2670	AsgA	A-signal production		Alpha Proteobacteria	S
				Cyanobacteria	
2778	PhoP2	DNA binding response regulator		Delta Proteobacteria	S
2779	PhoR2	Sensor histidine kinase		Delta Proteobacteria	S
2905	DofA	Hypothetical		Unique	
2913	AsgB	DNA binding protein		Delta Proteobacteria	S
3117	FruA	CsgA-dependent response regulator		Delta Proteobacteria	S
3204	RelA	Stringent response		Delta Proteobacteria	
3213	ActA	CsgA regulator		Delta Proteobacteria	S
3214	ActB	Sigma 54-dependent response regulator		Delta Proteobacteria	S
3225	FrgA	Polysaccharide export		Delta Proteobacteria	
3692	Nla18	Sigma 54-dependent response regulator		Delta Proteobacteria	S
3993	BsgA/Lon	ATP-dependent protease		Delta Proteobacteria	S
4016	Pfk1	6-phosphofructokinase		Delta Proteobacteria	E
4017	Pkn4	Ser/The protein kinase		Delta Proteobacteria	S
4042	Nla6	Sigma 54 dependent response regulator		Delta Proteobacteria	S
4138	FrzF	Protein methyl transferase		Delta Proteobacteria	S
4139	FrzG	Protein methyl esterase		Delta Proteobacteria	S
4140	FrzE	Chemotaxis histidine kinase		Delta Proteobacteria	S
4141	FrzCD	Chemotaxis receptor		Delta Proteobacteria	S
4142	FrzB	Hypothetical		Unique	
4143	FrzA	Hypothetical		Delta Proteobacteria	
4144	FrzZ	Response regulator		Delta Proteobacteria	S
4146	AldA	Alanine dehydrogenase		Firmicutes	E
4149	FrzS	Response regulator		Delta Proteobacteria	S
4486	FruE	Hypothetical	0.064	Unique	
4564	Esg	Branched chain keto acid dehydrogenase		Delta Proteobacteria	E
4565	Esg	Branched chain keto acid dehydrogenase		Delta Proteobacteria	E
4621	RfbC/SasA	Glycosyl transferase		Actinobacteria	E
				Firmicutes	
4778	PhoR1	Sensor histidine kinase		Delta Proteobacteria	S
4787	PhoP4	DNA-binding response regulator		Unclassified Proteobacteria	S
5123	MrpA	Sensor histidine kinase		Delta Proteobacteria	S
5124	MrpB	Sigma 54-dependent response regulator		Delta Proteobacteria	S
5125	MrpC	Transcription factor		Planctomyces	S
				Actinobacteria	
5766		TPR domain protein		Delta Proteobacteria	
5772	PilQ	Type IV pilus		Delta Proteobacteria	
5775	PilN	Type IV pilus		Delta Proteobacteria	
5776	PilM	Type IV pilus		Delta Proteobacteria	
5780	PilI	Efflux ABC permease		Delta Proteobacteria	
5786	PilC	Type IV pilus assembly protein		Delta Proteobacteria	
5788	PilB	Type IV pilus assembly ATPase		Delta Proteobacteria	
5870	SigE	Sigma factor		Delta Proteobacteria	
6307	FbdB	Chloride channel		Delta Proteobacteria	
6413	PhoP3	DNA binding response regulator		Delta Proteobacteria	S
6414	PhoR3	Sensor histidine kinase		Delta Proteobacteria	S
6692	DifE	Chemosensory histidine kinase		Delta Proteobacteria	S
6694	DifC	CheW-like coupling protein		Delta Proteobacteria	S
6696	DifA	Chemotaxis receptor		Delta Proteobacteria	S
6699	FbdA	Thiol oxidoreductase		Gamma Proteobacteria	E
6704		Acetyl transferase		Delta Proteobacteria	E
6855	MokA	Hybrid sensor kinase/response regulator		Delta Proteobacteria	S
6889	HthA	LuxR family transcriptional regulator		Delta Proteobacteria	S
6890	HthB	DNA-binding protein		Delta Proteobacteria	S
6996	AsgD	Sensor histidine kinase/response regulator		Delta Proteobacteria	S
7261	DevS	Hypothetical		Cyanobacteria	
7262	DevR	Hypothetical		Spirochetes	
7263	DevT	Hypothetical		Cyanobacteria	
				Spirochetes	
7324	FapA	Hypothetical		Unique	
7415	EpsZ	Glycosyl transferase		Delta Proteobacteria	E
7421	EpsV	Chain length determinant family protein		Delta Proteobacteria	
7422	EpsU	Glycosyl transferase		Chloroflexi	E
7433	EpsO	Von Willebrand factor type A domain protein		Alpha Proteobacteria	
7438	EpsK	Metal resistance protein		Gamma Proteobacteria	
7440	EpsI/Nla24	Sigma 54-dependent response regulator		Delta Proteobacteria	S
7441	EpsH	Glycosyl transferase		Alpha Proteobacteria	E
7445	EpsE	Glycosyl transferase		Alpha Proteobacteria	E
7448	EpsD	Glycosyl transferase		Delta Proteobacteria	E
7450	EpsB	Glycosyl hydrolase		Firmicutes	E
7451	EpsA	Glycosyl transferase		Delta Proteobacteria	E

1 Codon bias was determined using the software available at the Computational Microbiology Laboratory http://www.cmbl.uga.edu/software.html. The larger the number the greater the deviation from normal *M. xanthus* codon usage. Genes with no entries have a codon bias consistent with other *M. xanthus* genes.

2 tBLASTn algorithm was used to identify the four most closely related homologs. Each gene was classified as δ Proteobacteria if at least one of the four top homologs belongs to an organism in that group. Gene was classified as unique based on a 1e^−10^ cutoff. Gene was classified as ‘other’ if the closest four homologs belonged to another phylogenetic group.

3 Code refers to the classification category. Proteins known to be involved in signal production or sensory transduction are denoted with an ‘S’. The remaining genes were examined for a putative enzymatic function in the annotation, which is indicated by an ‘E’

Among the genes required for development very few exhibited a codon bias suggestive of LGT (Table 1). The exceptions include a unique gene, MXAN4486, and MXAN0581, which encodes the essential translation initiation factor InfC that is found in other δ Proteobacteria. However, the *M. xanthus* InfC protein is unique in that it contains a 66 amino acid C-terminal extension that is essential for development but not translation [18]–[20]. The precise function of this extension remains unknown, but is thought to aid in generating an essential developmental signal, the D-signal. We were unable to identify another InfC protein with a similar extension in the database.

While the remaining genes had a normal codon bias, the tBLASTn algorithm suggested that some were acquired by LGT because their closest relatives in the database were not members of the δ Proteobacteria (table 1). The contributions of LGT and vertical inheritance to the pool of genes for fruiting body development can be approximated from these data. 57 genes (73%) have a δ Proteobacteria phylogeny and a normal codon bias suggestive of vertical inheritance. 17 genes (22%) have either abnormal codon bias or produce protein products whose closest relatives are from another bacterial group. The remaining 2 genes (5%) have a normal codon bias but are unique and their phylogenetic source could not be identified. These results suggest that *M. xanthus* fruiting body development is not possible without alien genes.

When the genes were examined within the context of their function two striking correlations were observed. Genes encoding essential developmental enzymes were often acquired by LGT (table 1, 7/14, 50%). Most notably this includes genes required for exopolysaccharide (*eps*) and lipopolysaccharide (*rfb*) biogenesis. On the other hand most of the genes required for signal production and sensory transduction have a phylogeny rooted in the δ Proteobacteria and a normal codon bias (table 1, 37/41, 90%). Many of these genes have several paralogs in *M. xanthus* and seem to be products of duplication and divergence. These include two component systems, serine/threonine protein kinase systems, sigma 54-dependent response regulators, and chemosensory pathways [8].

**Figure 1:**
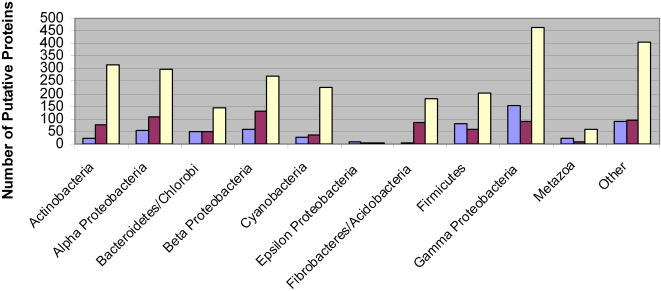
Taxonomic distribution of best normalized BLASTP matches outside the δ Proteobacteria. Bars indicate the number of best matches in a phylum or domain for *Myxococcus xanthus* (ivory bars), *Anaeromyxobacter dehalogenans* (magenta bars), and *Bdellovibrio bacteriovorus* (blue bars). Competitive matching was used for the detection of best hits using an E-value threshold of 10^−10^ and a minimum BLASTP score of 10^−10^

The phylogenetic sources of the alien developmental genes can suggest commonalities about the pool of donors. In some cases a clear gene source does not exist. For example, the MXAN4621 and MXAN5125 proteins have similar amino acid identities with proteins from widely different phyla (table 1). Nevertheless the phylogenic origin of each alien gene in the genome was explored systematically to determine whether any trends exist. The phyla containing the top four homologs of each alien gene are plotted in figure 1 for *Myxococcus*, *Anaeromyxobacter* and *Bdellovibrio*. Relative to the latter two organisms, *Myxococcus* has an unusually high abundance of genes whose closest relatives are found in the Actinobacteria and Cyanobacteria. Most soil Actinobacteria (like *Streptomyces*) are obligate aerobes. Cyanobacteria also live in aerobic habitats since they generate O_2_ during photosynthesis. These results suggest that *M. xanthus* acquired genes from organisms in aerobic habitats.

### Aerobic respiration

How alien genes are functionally integrated into existing pathways was examined with a less complex pathway, the electron transport chain. The nearly unique presence of aerobic respiration among the δ Proteobacteria, suggested that myxobacterial electron transport genes were derived by LGT. Examination of electron transport is also expected to reveal how recently acquired genes are integrated into functional networks. Was the entire pathway captured as a functional unit or were individual components acquired and used to replace existing components?

**Figure 2:**
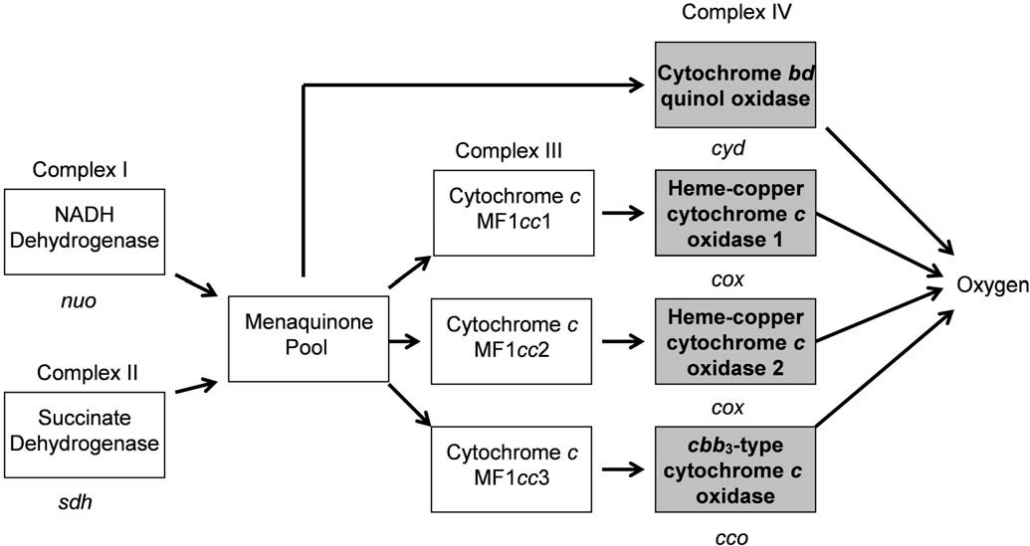
The *Myxococcus xanthus* electron transport chain. Phylogenetic trees were generated from individual Complex IV proteins (gray boxes). See text for detailed description.

**Figure 3:**
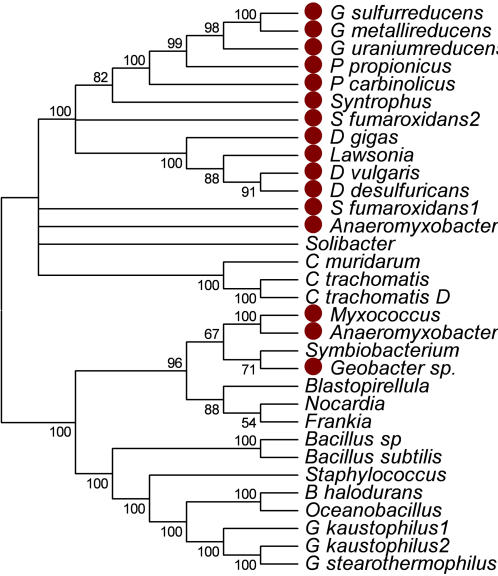
Neighbor-Joining Tree of the CydA Subunit of the predicted cytochrome *bd* quinol oxidase. The tree was generated by identifying the twenty most similar homologs in the non-redundant database at NCBI using the BLASTP algorithm as of Sept. 2006. Proteins were aligned using the Muscle algorithm. Bootstrap values and visualization were generated by the Mega 3.1 program. Tree branches were condensed for those with a bootstrap value >50%. Red filled circles are next to members of the δ Proteobacteria. *Anaeromyxobacter1-Anaeromyxobacter dehalogenans 2CP-C YP_464151.1; Anaeromyxobacter2-Anaeromyxobacter dehalogenans 2CP-C YP_466933.1; B_halodurans-Bacillus halodurans C-125; Bacillus_sp-Bacillus sp. NRRL B-14911; Bacillus_subtilis-Bacillus subtilis subsp. subtilis str. 168; Blastopirellula-Blastopirellula marina DSM 3645; C_muridarum-Chlamydia muridarum Nigg; C_trachomatis-Chlamydia trachomatis A/HAR-13; C_trachomatis_D-Chlamydia trachomatis D/UW-3/CX; D_desulfuricans-Desulfovibrio desulfuricans G20; D_gigas-Desulfovibrio gigas; D_vulgaris-Desulfovibrio vulgaris subsp. vulgaris str. Hildenborough; Frankia-Frankia sp. EAN1pec; G_kaustophilus1-Geobacillus kaustophilus HTA426 YP_147937.1; G_kaustophilus2-Geobacillus kaustophilus HTA426 YP_146457.1; G_metallireducens-Geobacter metallireducens GS-15; G_stearothermophilus-Geobacillus stearothermophilus; G_sulfurreducens-Geobacter sulfurreducens PCA; G_uraniumreducens-Geobacter uraniumreducens Rf4; Geobacter_sp.-Geobacter sp. FRC-32; Lawsonia-Lawsonia intracellularis PHE/MN1-00; Myxococcus-Myxococcus xanthus DK 1622; Nocardia-Nocardia farcinica IFM 10152; Oceanobacillus-Oceanobacillus iheyensis HTE831; P_carbinolicus-Pelobacter carbinolicus DSM 2380; P_propionicus-Pelobacter propionicus DSM 2379; S_fumaroxidans1-Syntrophobacter fumaroxidans MPOB ZP_00668206.1; S_fumaroxidans2-Syntrophobacter fumaroxidans MPOB ZP_00668271.1; Solibacter-Solibacter usitatus Ellin6076; Staphylococcus-Staphylococcus epidermidis ATCC 12228; Symbiobacterium-Symbiobacterium thermophilum IAM 14863; Syntrophus-Syntrophus aciditrophicus SB*

The protein components of the *M. xanthus* electron transport system were identified through comparative phylogeny. The upstream elements, NADH dehydrogenase (complex I) and succinate dehydrogenase (complex II), are common to many electron transport pathways (figure 2). The principal *Myxococcus* quinone is a menaquinone with 8 isoprenoid units [21], [22]. The δ Proteobacteria along with several other phyla lack the traditional *bc*_1_-complex III and contain a newly described complex III containing a five-heme cytochrome *c*, a one-heme cytochrome *c*, and a three-subunit molybdopterin oxidoreductase [23]. Genes encoding three copies of this complex, potentially one for each cytochrome *c* oxidase complex (denoted MF1*cc* in figure 3) are found in *M. xanthus* [23]. There are two broad classes of cytochrome oxidase, which reduces O_2_ to H_2_O (complex IV). Cytochrome *c* oxidase is coupled to the quinol:cytochrome *c* oxidoreductase (MF1*cc*) complex III. *M. xanthus* contains genes for three cytochrome *c* oxidases of this type, two *coxBAC* operons, and a *cbb*_3_ operon (table 2). The quinol oxidases derive electrons directly from the quinone pool and *M. xanthus* contains genes for both subunits of cytochrome *d* quinol oxidase (*cydAB*, table 2).

**Table 2:** Cytochrome oxidase proteins of *M. xanthus*.

Cytochrome *c* Oxidase				
Gene Name	CoxB	**CtaD (CoxA)**	CoxC	NA
Cytochrome c Oxidase Function	Subunit II	**Subunit I**	Subunit III	Subunit IV
MXAN number	3869	**3868**	3867	3866
Number of AA	346	**545**	207	121
MXAN number	6086	**6087**	6088	6089
Number of AA	348	**556**	222	151

Cytochrome *bd* Quinol Oxidase		
Gene Name	**CydA**	CydB
Cytochrome *c* Oxidase Function	**Subunit I**	Subunit II
MXAN number	**6912**	6913
Number of AA	**342**	445

Cytochrome *cbb*_3_ Oxidase					
Gene Name	FixG	CcoP	CcoQ	CcoNO	CcoS
Cytochrome *c* Oxidase Function	Fe-S Ferrodoxin	Subunit III	CcoQ Subunit	Subunit I/II	Maturation Protein
MXAN number	5538	5539	5540	5541	5542
Number of AA	479	176	60	793	63

**Figure 4:**
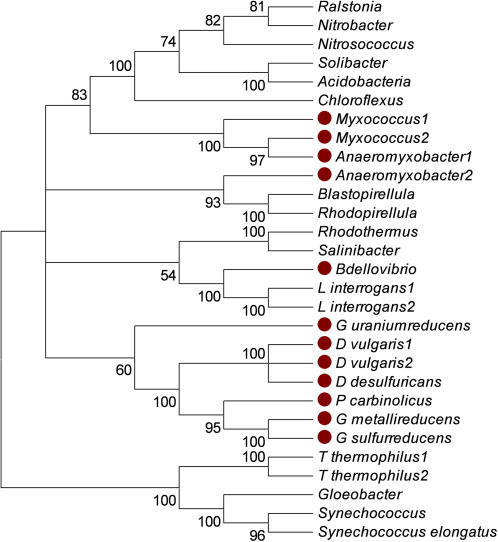
Neighbor-Joining Tree of Subunit I (CtaD or CoxA) of the predicted cytochrome *c* oxidase. The tree was generated by identifying the twenty most similar homologs in the non-redundant database at NCBI using the BLASTP algorithm as of Sept. 2006. Proteins were aligned using the Muscle algorithm. Bootstrap values and visualization were generated by the Mega 3.1 program. Tree branches were condensed for those with a bootstrap value >50%. Small filled circles are next to members of the δ Proteobacteria. *Acidobacteria-Acidobacteria bacterium Ellin345; Anaeromyxobacter1-Anaeromyxobacter dehalogenans 2CP-C YP_464016.1; Anaeromyxobacter2-Anaeromyxobacter dehalogenans 2CP-C YP_465481.1; Bdellovibrio-Bdellovibrio bacteriovorus HD100; Blastopirellula-Blastopirellula marina DSM 3645; Chloroflexus-Chloroflexus aurantiacus J-10-fl; D_desulfuricans-Desulfovibrio desulfuricans G20; D_vulgaris1-Desulfovibrio vulgaris; D_vulgaris2-Desulfovibrio vulgaris subsp. vulgaris str. Hildenborough; G_metallireducens-Geobacter metallireducens GS-15; G_sulfurreducens-Geobacter sulfurreducens PCA; G_uraniumreducens-Geobacter uraniumreducens Rf4; Gloeobacter-Gloeobacter violaceus PCC 7421; L_interrogans1-Leptospira interrogans serovar Copenhageni str. Fiocruz L1-130; L_interrogans2-Leptospira interrogans serovar Lai str. 56601; Myxococcus1-Myxococcus xanthus DK 1622 YP_632048.1; Myxococcus2-Myxococcus xanthus DK 1622 YP_634222.1; Nitrobacter-Nitrobacter hamburgensis X14; Nitrosococcus-Nitrosococcus oceani ATCC 19707; P_carbinolicus-Pelobacter carbinolicus DSM 2380; Ralstonia-Ralstonia eutropha JMP134; Rhodopirellula-Rhodopirellula baltica SH 1; Rhodothermus-Rhodothermus marinus; Salinibacter-Salinibacter ruber DSM 13855; Solibacter-Solibacter usitatus Ellin6076; Synechococcus-Synechococcus sp. PCC 7002; Synechococcus_elongatus-Synechococcus elongatus PCC 7942; T_thermophilus1-Thermus thermophilus HB27; T_thermophilus2-Thermus thermophilus HB8*

Trees are shown for enzyme subunits in bold in figures 3 and 4.

The origin of each *M. xanthus* electron transport chain gene was examined using both phylogenetic and compositional approaches. In all cases the codon usage was consistent with other highly expressed *M. xanthus* genes which is not surprising given that these are essential genes. However, the phylogenetic approach indicated that the succinate dehydrogenase proteins SdhA (MXAN3539), SdhB (MXAN3540), and SdhC (MXAN1072), which together form complex II, are most similar to their counterparts in aerobic Firmicutes and Actinobacteria and are not present in any other δ Proteobacteria (not shown). Furthermore, the cytochrome oxidase genes (complex IV) were acquired from diverse phylogenetic sources. In contrast, NADH dehydrogenase (complex I) and the MF1*cc* quinol:cytochrome *c* oxidoreductase (complex III) are most similar to their counterparts in other δ Proteobacteria. These results argue that the electron transport pathway is encoded by a patchwork of genes, some inherited vertically and others acquired from diverse phylogenetic sources.

Cytochrome oxidase genes have been subject to intense scrutiny because of their dissemination by LGT over vast phylogenetic distances [7]. The distribution of one subunit from each cytochrome oxidase was examined (bold subunits in table 2). Among the top twenty BLASTP hits to the *M. xanthus* cytochrome *cbb*_3_ oxidase, *Bdellovibrio* is the only δ Proteobacteria member (not shown) suggesting that this *cbb_3_* oxidase was acquired by the only two aerobic members of this group.

A neighbor-joining tree of the CydA subunit of the cytochrome *d* quinol oxidase revealed two versions of this operon within the δ Proteobacteria (figure 3). One is found in all δ Proteobacteria with the exception of *M. xanthus* and is presumed to be ancient and vertically transmitted. *M. xanthus* appears to have lost this operon sometime after its divergence from *A. dehalogenans*. It is surprising to find this oxidase in anaerobic organisms where O_2_ is highly toxic [24]. A distinctly different *cydAB* operon is found in *A. dehalogenans* and *M. xanthus* and was likely incorporated by LGT prior to the divergence of the two organisms (figure 3). The *M. xanthus cydAB* operon falls in the same clade as diverse phylogenetic groups, most notably Actinobacteria and Planctomycetes.

The *coxBAC* operon is found in many members of the δ Proteobacteria (figure 4). Again, the presence of this operon is surprising for anaerobic species where the function is not known and suggests an ancient origin in the δ Proteobacteria. In *M. xanthus* and *A. dehalogenans* the *coxBAC* operon has a different phylogenetic history from that of the other δ Proteobacteria and falls in the same clade as an eclectic mixture of organisms from diverse phyla. The results are consistent with the idea that *Myxococcus* and *Anaeromyxobacter* have lost the ancient version of this operon and acquired a different one. In addition, the *coxBAC* operon has been duplicated in *M. xanthus*.

In summary, the *M. xanthus* electron transport chain is a chimeric pathway containing components from diverse phylogenetic sources. *M. xanthus* has lost ancestral cytochrome oxidase genes common to other δ Proteobacteria and acquired other cytochrome oxidase genes. Acquisition of novel cytochrome oxidase and succinate dehydrogenase genes is coincident with the appearance of aerobic growth. Curiously, the only other aerobic organism in the δ Proteobacteria, *Bdellovibrio*, has notable differences from *Myxococcus* suggesting that it acquired aerobic metabolism using different gene sources.

## Discussion

The genome of virtually every free-living bacterial species contains a contingent of genes acquired by LGT [4]. Genes acquired by LGT can provide selective advantages with regard to antibiotic resistance, carbon utilization, and habitat range [4], [6], [7]. Indeed, most recent changes to the metabolic network of *E. coli* are due to LGT rather than gene duplication [25]. We estimate that roughly a quarter of the *Myxococcus* genome has been acquired by LGT based on phylogeny and/or codon usage. Some alien genes came from pathways that are found in diverse phyla, for example succinate dehydrogenase and cytochrome oxidase, and their import would be expected to provide a strong selective advantage by allowing aerobic growth. Other alien genes were fashioned into a unique developmental cycle and presumably played a different role in their previous host.

### Developmental innovation is correlated with a habitat shift

What event(s) facilitated the innovation of this unique developmental cycle and the divergent evolution of Myxococcales from other δ Proteobacteria? We propose that the ancient myxobacterium gained access to a novel gene pool that was not available to other δ Proteobacteria. A phylogenetic analysis of >220,000 proteins from the genomes of 144 prokaryotes identified specific ‘highways’ of lateral inheritance, some among distantly related organisms that live in similar environments [26]. The succinate dehydrogenase and cytochrome oxidase genes were clearly acquired from aerobic organisms since they mediate aerobic respiration. Over 500 *M. xanthus* proteins have as their closest relatives proteins from Actinobacteria and Cyanobacteria (figure 1). This number exceeds by 10-fold the number of Actinobacteria and Cyanobacteria homologs found in *Bdellovibrio*. Most soil Actinobacteria, like *Streptomyces*, are aerobes and Cyanobacteria make their habitat aerobic by performing oxygenic photosynthesis. These factors argue that a shift to an aerobic habitat has given the Myxococcales access to a novel gene pool via LGT. Whether this habitat shift predated the emergence of fruiting body development is not clear but this issue could potentially be resolved when the genome sequences of more δ Proteobacteria are completed.

### *Myxococcus* electron transport is a chimeric pathway

Alien genes are generally integrated at the periphery of metabolic networks [25] where they could provide an immediate selective advantage. Multiple terminal electron acceptors provide flexibility in the face of ever changing environmental stresses. Of the four new *M. xanthus* terminal oxidase complexes, one involved the addition of a new oxidase (*cco*; *cbb3*-type cytochrome oxidase), one involved replacement of an ancient oxidase (*cyd*; cytochrome *bd* quinol oxidase), and one involved replacement of an ancient oxidase followed by duplication of the imported operon (*cox*; heme-copper cytochrome *c* oxidase). O_2_ has one of the most positive reduction potentials of any terminal electron acceptor (O_2_/H_2_O = 0.82 V) so these cytochrome oxidases are located at the periphery of the electron transport chain. Succinate dehydrogenase (complex II) is located at the other end of the electron transport chain where it, along with NADH dehydrogenase (complex I), provide reducing equivalents. Succinate dehydrogenase catalyzes the interconversion of succinate and fumarate with the reduction of FAD allowing an organism to harvest the full potential of the tricarboxylic acid (TCA) cycle. Succinate dehydrogenase transfers reducing equivalents derived from the TCA cycle to the electron transport chain. In organisms with mixed respiration strategies like *E. coli* succinate dehydrogenase is produced only during aerobic growth [27]. The phylogenetic sources for the *M. xanthus* dehydrogenase and oxidases are different suggesting a piecemeal integration into the electron transport pathway.

### Predation as a means of acquiring genes

In a microcosm containing ^13^C labeled *E. coli* added to agricultural soil, the heavy carbon isotope was enriched in wild myxobacteria indicating that myxobacteria are predators [28]. Does predation enhance the acquisition of new genes by the predator? The LGT observed with *Bdellovibrio* has been proposed to arise, at least in part, through the predatory capabilities of the organism [13]. Indeed, the *Bdellovibrio* genome is enriched in genes whose closest homolog is in the γ Proteobacteria, the group containing the principal prey species [13]. Like *Bdellovibrio,* the *M. xanthus* genome is enriched in genes whose closest homolog is in the γ Proteobacteria, the group containing the commonly used prey species *E. coli* [28]. Myxobacteria have a much broader prey range than *Bdellovibrio* and significantly, a broader distribution of closest gene homologs (figure 1). Some notable differences from *Bdellovibrio* include genes from Actinobacteria, Acidobacteria, and Cyanobacteria (i.e. >8-fold increase), which tend to be residents of same type of soil habitats [29]. Acidobacteria are thought, on the basis of 16S gene abundance, to be one of the most prominent members of soil, though few species have been cultivated [30]. It has been recently proposed that Acidobacteria form a sister clade with the δ Proteobacteria [31]. While vertical inheritance from a common ancestor could result in the high representation of Acidobacteria genes, this hypothesis does not explain why *Bdellovibrio* has so few Acidobacteria genes (figure 1). Organisms in the other highly represented bacterial groups are known to be excellent food sources for *M. xanthus* in the laboratory [32]. *M. xanthus* is known to acquire genes from *E. coli* by conjugation [33] and transduction [34] under laboratory conditions but gene transfer in the natural environment has not been experimentally demonstrated.

### Conclusions and prospectus

Our results argue that genes acquired from community members influence bacterial evolution. Successful community members may foster the evolution of successful communities by sharing genetic and phenotypic innovations that promote fitness. Cooperative evolution can have strong selective advantages in nature as demonstrated by the widespread emergence of antibiotic resistance or LGT of terminal oxidases in electron transport chains. It follows then that biologically diverse habitats aid the evolution of new bacterial species by providing a larger pool of prospective genes. While studies of the biogeography of microorganisms is in its infancy, bacterial communities can be endemic to certain areas or associated with unique species of higher organisms [35]. Habitat destruction and loss of species diversity could restrict the evolution of new bacterial groups by limiting combinations of LGT.

## Materials and Methods

### Isolation of fruiting body deficient mutants

*Myxococcus xanthus* LS2208 (Δ*fibA*) cells were grown to a density of 5×10^8^ cells/ml in CYE broth [10 g/L Difco Casitone, 5 g/L yeast extract, 10 mM 3-(*N*-morpholino) propanesulfonic acid (MOPS; pH 7.6), and 4 mM MgSO_4_]. A 3 ml aliquot was harvested by centrifugation, washed twice with 1 ml of sterile distilled water, and resuspended in 30 µl sterile distilled water. Washed cells were mixed with plasmid pMycoMar [14], and electroporated at 0.65 kV, 400 Ω and 25 µF [36]. The electroporation cuvette was flushed immediately with 1 ml of CYE broth to recover cells, which were then incubated with shaking at 32°C for 4 h before plating on CF agar [10 mM Tris HCl, pH 7.6, 1 mM KH_2_PO_4_, pH 7.6, 8 mM MgSO_4_, 0.2 mg/ml, (NH_4_)_2_SO_4_, 150 µg/ml casitone, 1 mg/ml Na-pyruvate, 2 mg/ml Na-citrate, 1.5% Difco agar] supplemented with kanamycin (50 µg/ml) (CF Km) and incubated at 32°C. After 9–10 days, plates were screened under the microscope to identify colonies that were defective in making fruiting bodies. Potential mutants were screened again on CF Km plates and once on TPM agar [10 mM Tris HCl, pH 7.6, 1 mM KH_2_PO_4_, pH 7.6, 10 mM MgSO_4_, 1.5% Difco agar]. Approximately 40,000 colonies were screened. Strains containing a *magellen*-4 insertion were backcrossed to *M. xanthus* DK1622 (wild type) by electroporation of 1 µg genomic DNA [37] or generalized transduction with phage Mx4 [38].

### Cloning of *M. xanthus* genomic DNA flanking *magellan*-4 insertions

To clone *magellan-4* insertions, genomic DNA was isolated from vegetative cultures grown in CYE medium containing 50 µg/ml kanamycin. A 1 ml cell culture was harvested by centrifugation and resuspended in 0.2 ml of 1X PBS buffer [8 g NaCl, 0.2 g KCl, 1.44 g Na_2_PO_4_ and 0.24 g KH_2_PO_4_ in 1000 ml distilled H_2_O, pH 7.4]. Genomic DNA was isolated by using Invitrogen Easy-DNA kit. Genomic DNA (0.5 µg) was digested with BssHII (New England Biolabs) in a total volume of 20 µl and digestions were dialyzed on a 0.025 µm pore size filter (Millipore) against distilled water for 30 min (drop dialysis). 8 µl of this DNA was treated with T4 DNA ligase (Promega) and drop-dialyzed before electroporation into *E. coli* host CC118 [15]. Electroporants were recovered on LB agar containing kanamycin (50 µg/ml) after incubation at 37°C for 24 h. Plasmid DNA was sequenced with primers Mar1 or Mar2 [15].

### Fruiting body formation and sporulation assay

*Myxococcus xanthus* cells were grown in CYE broth to about 5×10^8^ cells/ml. Cells were resuspended in TPM buffer [10 mM Tris HCl, pH 7.6, 1 mM KH(H_2_)PO_4_, pH 7.6, 10 mM MgSO_4_] to a final density of 1×10^10^ cells/ml. 10 µl of each suspension was spotted onto TPM agar plates and incubated at 32°C. Digital images of fruiting body formation were taken every 24 h for a total of 72 h.

Cells were resuspended in 1 ml of TPM buffer, heated at 50°C for 2 hours, and sonicated to kill vegetative cells. Spore production was determined by direct counts using a Petroff-Hauser chamber. Spore viability was determined by plating serial dilutions on CYE agar plates. Plates were incubated at 32°C for 5 days before counting colonies.

### Genomic analyses

Phylogenetic and comparative genomic analyses were conducted with data from >300 genomes. Predicted peptides from each of eight completed δ Proteobacteria genomes were obtained from NCBI in March and April 2006 including *Anaeromyxobacter dehalogenans, Bdellovibrio bacteriovorus, Desulfotalea psychrophila, Desulfovibrio desulfuricans, Geobacter sulfurreducens, Lawsonia intracellularis, Myxococcus xanthus, and Pelobacter carbinolicus.* Comparison of peptides was determined using the BLASTP algorithm without filtering against the non-redundant peptide database at NCBI during this time period [39]. The top ten hits for each predicted peptide were identified with an E value<10^−10^. The species of each top hit and the corresponding bacterial group were identified from the BLAST algorithm output. The identity of each bacterial group used the taxonomic data provided by NCBI. Alignments of predicted peptides were generated using the Muscle algorithm [40]. Neighbor-Joining trees and tree visualization were performed using MEGA version 3.1 [41]. Tree branches with a bootstrap value<50% were condensed.
